# Efficacy of throwing exercise with TheraBand in male volleyball players with shoulder internal rotation deficit: a randomized controlled trial

**DOI:** 10.1186/s12891-020-03414-y

**Published:** 2020-06-13

**Authors:** Mohsen Moradi, Malihe Hadadnezhad, Amir Letafatkar, Zohre Khosrokiani, Julien S. Baker

**Affiliations:** 1 Faculty of Physical Education and Sport sciences, Department of Biomechanics and Sport injuries, Kharazmi University, Tehran, Islamic Republic of Iran; 2 Department of Biomechanics and Sport injuries, Kharazmi University, Tehran, Islamic Republic of Iran; 3 Biomechanics and Corrective Exercise Laboratory, Faculty of Physical Education and Sport sciences, Kharazmi University, Mirdamad Blvd., Hesari St, Tehran, Iran; 4 Centre for Health and Exercise Science Research, Hong Kong Baptist University, Kowloon Tong, Hong Kong

**Keywords:** Glenohumeral internal-rotation deficit, Injury prevention, Volleyball player

## Abstract

**Background:**

The Glenohumeral internal-rotation deficit (GIRD) is related to the altered eccentric external-rotator (ER), the concentric internal-rotator (IR), muscle strength, and the ER: IR ratio. GIRD has been documented as a risk factor for shoulder injuries. However, few studies have investigated the effect of an exercise training on these parameters in athletes with GIRD. Therefore, the purpose of this study was to evaluate the effects of an 8-week throwing exercise with a TheraBand for retraining the rotator cuff on Electromyography (EMG) activity of selected muscles, rotator cuff muscle strength, the glenohumeral (GH) joint IR range of motion (ROM) and GH joint position sense in asymptomatic male volleyball players with GIRD.

**Methods:**

Sixty male volleyball players with GIRD were randomized into either a training group or a control group. The experimental group underwent an 8-week throwing exercise with a TheraBand including 5 sessions of stretching and 3 sessions of strengthening exercises per week. The control group received an active self-exercise program. EMG (onset time and muscle activation), shoulder range of motion (ROMs), strength and GH joint position sense were all assessed pre and post trainings.

**Results:**

There were statistically significant within-group differences in the EMG activity of the anterior deltoid (*p* = 0.005), middle deltoid (*p* = 0.007), posterior deltoid (*p* = 0.004), infraspinatus (*p* = 0.001) and supraspinatus (*p* = 0.001) muscles, IR ROM (*p* = 0.001), rotator cuff muscle strength ratio (*p* = 0.001), and GH joint position sense (*p* = 0.033) in the experimental group. A 2 × 2 analysis of variance with a mixed model design and independent and paired t-tests were used for statistical analysis.

**Conclusions:**

Throwing exercise with a TheraBand improved shoulder muscle activation, IR ROM, rotator cuff muscle strength ratio and GH joint position sense in participants with GIRD. These findings may improve the treatment of GIRD in a clinical setting. Although the results are significant, further studies should follow up the long-term effects of the Throwing exercise with a TheraBand on GIRD.

**Trial registration:**

Current Controlled Trials using the UMIN-RCT website with ID number of, UMIN000038416 “Retrospectively registered” at 2019/10/29.

## Background

Overhead athletes, like volleyball players, can demonstrate altered glenohumeral (GH) joint mobility and flexibility in the dominant hand resulting in significantly less internal rotation (IR) and greater external rotation (ER) of the shoulder, classified as glenohumeral internal rotation deficit (GIRD) [1–5]. Two different types of GIRD have been described, anatomical and pathological. Anatomical GIRD has been described as a loss of less than 18–20° deficit of glenohumeral IR with symmetrical total rotational motion (e.g., sum of ER + sum of IR) of the uninvolved shoulder within 5°. Pathological GIRD has been identified as a loss of glenohumeral internal rotation greater than 18–20° with a corresponding loss of the total rotation greater than 5° seen in the throwing shoulder compared to the non-throwing shoulder, respectively [1, 6].

In a recently published study, a decrease in ER strength in combination with GIRD was associated with shoulder problems in handball players [4]. However, there has been no specific research demonstrating how GIRD affects the ER:IR strength ratio in adolescent overhead athletes. Moreover, the excessive posterior capsular tightness produced by repetitive functional positions of 90° shoulder abduction and ER ≥ 90° makes the humeral head shift antero-superiorly compared with a normal shoulder in throwing movements [5, 7].

These adaptations could alter muscle stiffness (posterior deltoid, subscapularis and teres minor), glenohumeral (GH) joint flexibility, posterior capsular stiffness, rotator cuff ER:IR strength ratio and joint position sense in the dominant shoulder [7–9].

Normative data of eccentric and concentric peak strength of ER and IR, as well as functional ratios are available for various groups of athletes (3, 10–13) and a ratio lower than 0.76 is an accepted risk factor for shoulder injuries. Studies report that athletes with GH joint instability and impingement syndrome have lower ER:IR ratios (9,14) and the lower ratios increase shoulder pathologies in those athletes (9,11,14).

Moreover, following postero-inferior capsular tightness (with resultant GIRD), GH contact pressure and rotator-cuff impingement significantly increase [1]. These abnormalities can lead to changes in the activities of the rotator cuff and deltoids in GIRD resulting in a significant reduction in rotator cuff ability to centralize the humeral head and to counterbalance the deltoid upward shear force [1, 10].

As a result, for optimal performance in overhead athletes, the primary focus should be on stretching capsular tightness, balancing the agonist and antagonist muscle strength around the scapula and shoulder, providing dynamic GH joint stability, restoring shoulder muscle activation and altering the ER: IR strength ratio [1, 10–14].

While throwing motions may be similar in some throwing sports, the athletes adapt to the demands of the sports differently and specifically; thus, stretching/strengthening programs designed for an athlete in a specific sport may not be the most effective program for all athletes to mitigate their symptoms [6]. Most studies have supported the effectiveness of stretching or strengthening exercises on IR range of motion (ROM), shoulder pain and scapular mechanics in overhead athletes with or without impingement symptoms and GIRD [1, 7, 11, 13, 15]. However, studies reported the importance of evaluating the shoulder muscles especially the rotator cuff in overhead athletes with joint instability and shoulder motor control issues (1,11,20). Few studies have assessed the EMG activity of the rotator cuff, deltoid muscles and ER:IR strength ratio after an 8-week throwing exercise with a TheraBand in people with GIRD. Further research is needed to better evaluate the effectiveness of an exercise intervention on the symptoms of GIRD.

Therefore, the purpose of this study was to investigate the effects of an 8-week throwing exercise with a TheraBand for retraining the rotator cuff muscles in asymptomatic male volleyball players with GIRD. Evaluation included EMG activities of selected muscles, rotator cuff muscle strength, GH joint IR ROM and GH joint position sense. We hypothesized that an 8-week throwing exercise with a TheraBand could restore the activation of the deltoid and rotator cuff muscles. We also hypothesized that these exercises would improve the ER: IR strength ratio, IR ROM and GH joint position sense.

## Methods

### Study design

The experimental protocol used was a pragmatic, 2-arm, parallel-designed, randomized, controlled, assessor-blinded clinical trial. This study was registered at (UMIN-RCT), and the clinical trial registration of the study was enrolled under the number (UMIN000038416).

### Participants

A sample size calculation was performed based on data from a pilot study using G*power (v3.1.9.2, Heinrich-Heine-University, Dusseldorf, Germany). Using an ANOVA test with 2 groups and 2 test sessions, a power of 0.95, an ɑ of 0.05, and an effect size of 0.50 a total sample size of 54 patients was needed. As a result, a total of 60 male participants were enrolled in the study (30 per group) to allow for a 10% dropout rate.

Ninety participants were assessed for eligibility. Sixty university male participants (volleyball players) with GIRD were considered eligible to participate in the study based on the study inclusion criteria. They were randomly assigned by the slot-drawing method into an experimental group and a control group. The study was approved by the local ethics committee at Kharazmi University. Prior to enrolment in the project, participants signed an informed consent form outlining ethical standards in accordance with the Declaration of Helsinki.

Participants with GIRD were diagnosed (the IR and ER ROM were assessed using a goniometer) as the difference in IR ROM greater than 18° with a corresponding loss of total rotational motion greater than 5° when compared bilaterally [6, 10]. Inclusion criteria were the presence of GIRD, and male volleyball players with regular volleyball training (three sessions per week and 90 min for each session) [16].

The exclusion criteria included a history of GH dislocation in the past year, structural abnormalities in the shoulder and thoracic regions such as scoliosis and kyphotic postures, any surgery in the upper limb region in the past two years, participating in shoulder rehabilitation in the past year, neurological and musculoskeletal disorders that limit movement, and pain in the upper limb prior to and after the tests [10].

### Procedures

Muscle activity, muscle strength, GH IR range of motion, and GH joint position sense were assessed. All tests were performed by the same examiner blinded to the group assignments and previous measurements. All treatments were supervised by an expert physiotherapist.

In addition to their routine exercises, participants in the experimental and control groups performed their off-season exercises. The experimental group underwent the throwing exercise with a TheraBand for approximately 40 min per session, three sessions a week lasted eight weeks. Each session also consisted of a 10-min warm up (running, stretching exercises) and a 5-min cool down. Data were collected before and after the prescribed exercise program, using the same procedure. The control group underwent a home self-exercise program for three 40-min sessions/week for 8 weeks, referring to the medical center every week as necessary. The control group did not perform any strengthening exercises but performed stretching exercises (Table 1).

**Table 1:** The throwing exercise with TheraBand

Exercises		Position/description
Eccentric exercise for the external rotators in an abducted position Weeks (repetition _*_ time)	Performed by the experimental group	Exercises that accentuate the eccentric phase and “avoid” the concentric phase in order to load the muscles based on their eccentric capacity. Figures shows an example of an eccentric exercise for the external rotators in general in an abducted position. Week 1–2: (3* 15 s) Week 3–6: (3* 17 s) Week 7–8: (3* 20 s)
External Rotation 90 Weeks (repetition _*_ time)	Performed by the experimental group	Week 1–2: (3* 15 s) Week 3–6: (3* 17 s) Week 7–8: (3* 20 s)
Perturbations applied to the patient’s extremity in the 90/90 position using the scapular plan Weeks (repetition _*_ time)	Performed by the experimental group	Perturbations applied to the patient’s extremity in the 90/90 position using the scapular plane. Week 1–2: (3* 15 s) Week 3–6: (3* 17 s) Week 7–8: (3* 20 s)
Catching exercise Weeks (repetition _*_ time)	Performed by the experimental group	Prone 90/90 plyometric exercise for posterior rotator cuff and scapular training. Week 5–6: (3* 10 s) Week 7–8: (3* 12 s)
Sleeper Stretch 1 Weeks (repetition _*_ time)	Performed by the experimental group	On the prone position Week1–8: (5* 30 s)
Sleeper Stretch 2 Weeks (repetition _*_ time)	Performed by the experimental group	*Performed in prone with manual stabilization of the scapula.* Week1–8: (5* 30 s)
Sleeper Stretch 3 Weeks (repetition _*_ time)	Performed by the experimental group and control group	*Traditional position, self-stretch performed in side lying with arm at 90 degrees of abduction.* Week1–8: (5* 30 s)
Sleeper Stretch 4 Weeks (repetition _*_ time)	Performed by the experimental group and control group	Alternate sideling position, self-stretch with arm elevated above 90 degrees. Week1–8: (5* 30 s)
Sleeper Stretch 5 Weeks (repetition _*_ time)	Performed by the experimental group and control group	alternate sideling position, self-stretch with arm at 45 degrees Week1–8: (5* 30 s)
Sleeper Stretch 6 Weeks (repetition _*_ time)	Performed by the experimental group	*Passive stretch performed in supine, with stabilization of the scapula.* Week1–8: (5* 30 s)

The prescribed throwing exercise with a TheraBand for the experimental group and recommendation to control posture and performance (Table 1) were given to the control group following completion of the study.

### IR ROM assessment

**Fig. 1:**
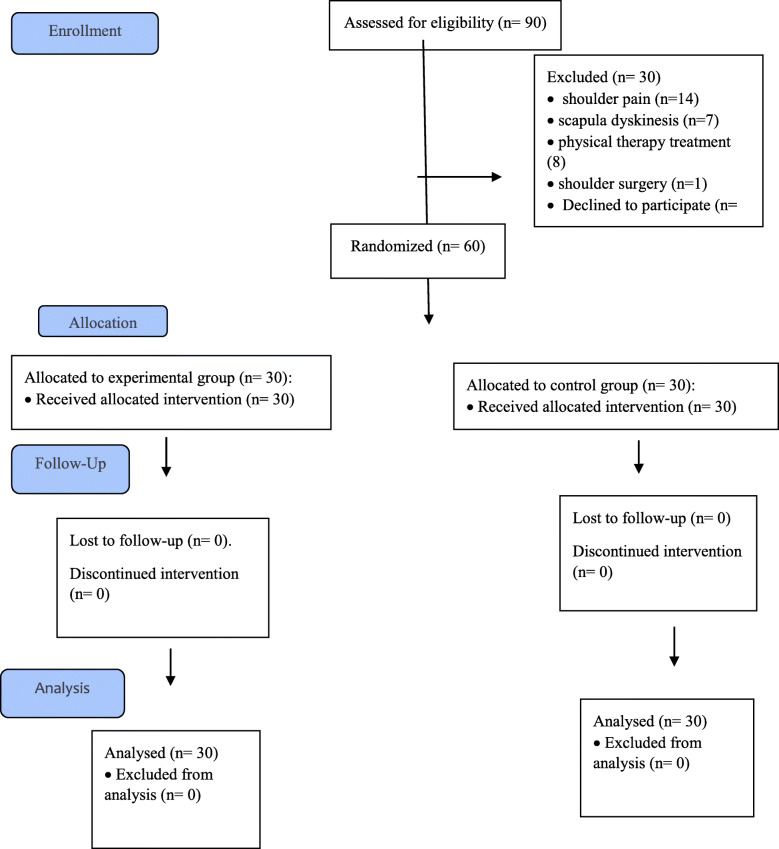
Consort flow diagram

A goniometer was used as a reliable (ICC = 0.94) tool to measure IR ROM of the GH joint positions [10]. To evaluate IR ROM, in a supine position, the participant was told to relax the shoulder girdle muscles. The dominant shoulder was placed at 90 degrees of abduction, and the elbow at 90 degrees of flexion. Then, the first examiner moved the shoulder joint passively into IR and placed the other hand on the subject’s acromioclavicular joint. Then, as soon as the motion in the acromioclavicular joint was noted, the IR motion was stopped [10], and the participant’s hand was kept constant by the second examiner. The IR ROM around the coronal axis was then calculated (Fig. 1). To increase the accuracy of the measurement and reduce any error, the mean value of three trials was used in data analysis [10].

### EMG muscle activity

**Fig. 2:**
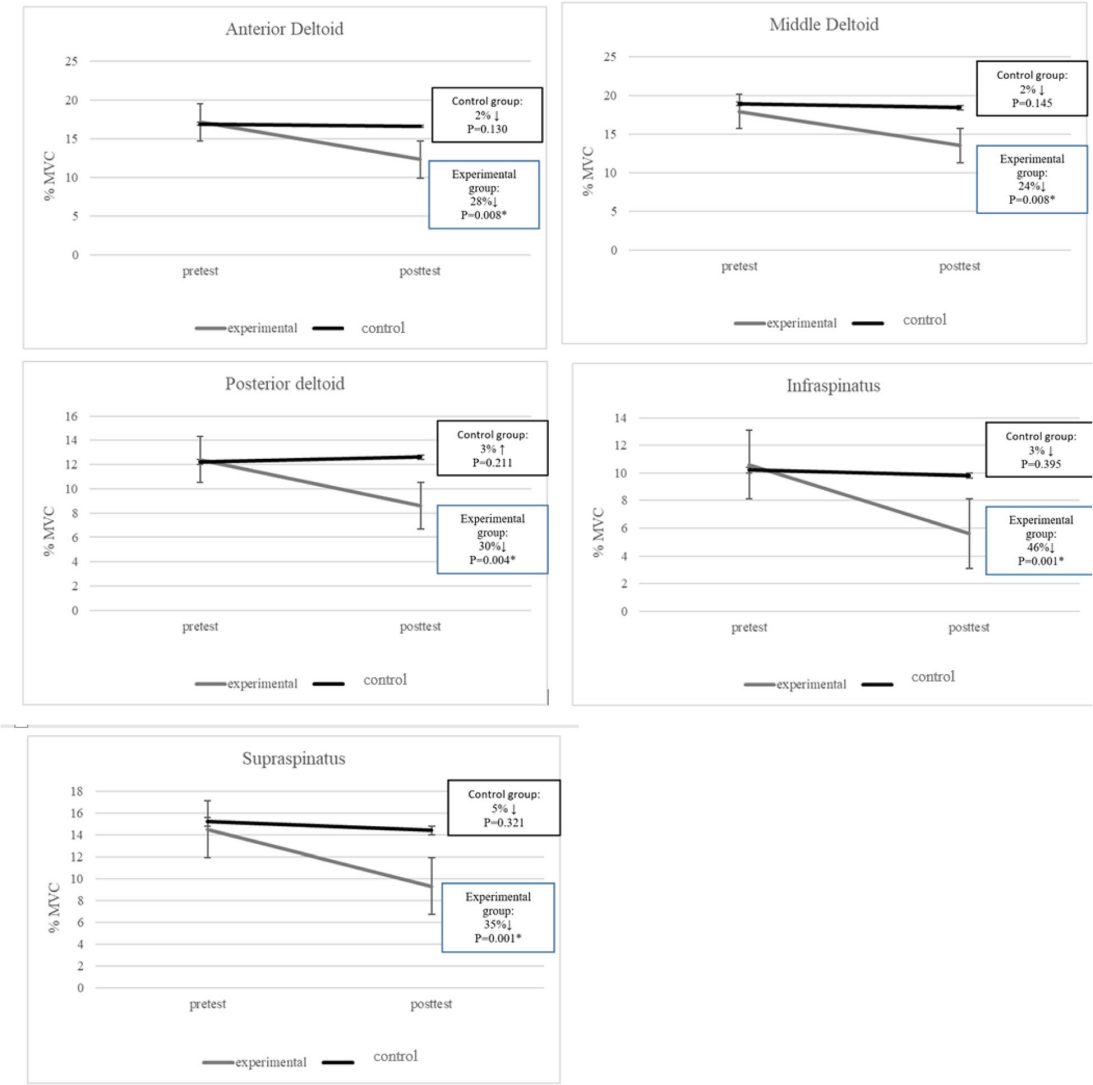
Muscle activation (%MVC) alteration from pretest to post-test

Surface Electromyography (EMG) (MIE model, England) was used to record muscle activity (Fig. 2). After skin preparation, bipolar electrodes (Ag/AgCl rectangular F-RG1 paired bipolar disposable surface electrodes (SKINTACT, Austria) were adhered with a 2 cm inter-electrode distance. The surface EMG of the infraspinatus, supraspinatus, posterior, medial and anterior deltoid muscles were recorded. A researcher who had experience with the procedure of placing electrodes was careful to palpate the anatomical landmarks to ensure the correct placement according to the method of electrode placement based on the SENIAM method [17]. EMG signals were amplified (ten times) and filtered (between 20 and 500 Hz). For normalization of the data, the root mean square was divided into the maximum voluntary contraction (MVC) of each muscle. MVC was recorded on two occasions and each measurement needed 5 s to complete. The first and last seconds of recorded during data collection were removed and the mean of the three seconds was used to normalize the data [18]. The highest EMG signal amplitude obtained for each muscle during the MVC testing procedure (described below) was used for normalization. The highest EMG signal was defined as the highest mean RMS value obtained over a consecutive 3-s period of the MVC test [15]. Following MVC testing, participants were instructed to perform three arm abductions while the arm was beside the trunk, with the elbow at 0° and the hand in a neutral position. Participants were required to move to 90° abduction in the horizontal plane with complete humeral internal rotation. The speed of the movement was controlled by a metronome, with each elevation performed using a four-second ascending and a four-second descending methodology. The participants could practice for three to five trials to familiarize themselves with the motion prior to data collection [15].

Onset time of a muscle was visually identified and determined using an algorithm (Matlab 7.1) as the midpoint of the first 25 ms moving window in which the average muscle activity was greater than three standard deviations above the baseline measure [19]. Considering both negative and positive influences of different parameters, Hodges and Bui (1996) identified that the low pass filtering of an EMG signal at 50 Hz using a moving window of 25 ms is the value that the mean should exceed. A threshold of three standard deviations above baseline accurately represents the time of the onset of EMG activity [18, 19]. Additionally, using these parameters, this method of evaluating the onset of activation timing has been shown to be highly repeatable with an intraclass correlation of 0.91 (95% CI 0.67–0.98). The initial activation timing was then calculated as the time from the start of movement to initial muscle activation, with negative values indicating activation prior to the start of the movement. Group mean (95% CI) initial activation timing was calculated for each muscle at each load. We calculated the EMG onset of all shoulder muscles relative to that of the prime mover, the deltoids [18].

All EMG data was imported into MATLAB (version R2013b; The MathWorks, Natick, MA) were full-wave rectified, and the electrocardiogram data were removed from the EMG data using a modified turning-point filter [11].

### Rotator cuff muscle strength assessment

In this study, a 90 k/s isokinetic dynamometer (IsoMed2000 D&R GmbH, Germany) was used to measure the isokinetic strength of the rotator cuff muscles of the dominant shoulder. The participants sat on the dynamometer seat. The trunk was fixed with straps. The dominant shoulder was placed on the scapular plan (45° abduction, 30° flexion, 90° flexion of the elbow and forearm in pronation). This position was set as anatomical zero and the range of movement was set at 90 degrees for total shoulder IR and ER at 90°/s angular velocity, by removing gravity effects. The concentric and eccentric contractions of the muscles were measured with 30 s rest observed between the trials. For accurate measurement, three sub-maximal muscle strength trials were performed as warm-up for both muscle groups before the test, with a 5-min recovery between warm-up and tests. The mean of three maximum strength trials was considered as a valid measure [20]. Functional ER: IR strength was calculated as maximal eccentric ER strength divided by maximal concentric IR strength [15]. The shoulder and elbow were positioned in the overhead throwing position (the shoulder abducted to 90°, and the elbow flexed to 90°) [21]. An experienced therapist (M.H.) supervised isokinetic strength testing. During the test, verbal encouragement was given to the participants to exert maximum muscular effort.

### Joint position sense measurement

An isokinetic device was used (Biodex 3) to measure GH joint position sense. The participants were informed about the procedure in the test 24 h before collecting the first data set, followed by a practical session. Before testing, each participant underwent a 10-min warm-up consisting of stretching exercises. Both shoulders were tested, with the uninvolved shoulder tested first. To perform the joint position sense test, with the arm in 90° abduction and 0° rotation on the horizontal plane with 90° flexion of the elbow, the participant was asked to move the arm into the 45° internal rotation. To minimize the involvement of other muscle groups or unnecessary movement, the trunk was fixed using a strap. The starting position for angle repositioning of 90 degrees was a 45-degree angle, and for angle repositioning of 45 degrees the starting position was a 90-degree angle. For accurate measurement, two practice trials were performed before data collection [16].

Data collection commenced when the participant, eyes being covered by a blindfold, was asked to move the arm from the starting position to a target angle (45 or 90 degrees). When the participant felt the target angle, he stopped the movement. This procedure was repeated three times, and the difference between the angle recorded and the target angle was recorded as an error (absolute angular error). The mean of three trials was used in statistical analysis [16].

### Intervention

The protocol used in this study was based on the study of Laudner et al. (2008) and Ellenbecker et al. (2010) and comprised of an 8-week throwing exercise with a TheraBand including 5 sessions of stretching and 3 sessions of strengthening exercises per week. Each session lasted about 30 min [7, 21]. Exercises included eccentric exercises for ER in shoulder abduction, ER in 90 degrees, catching exercises and six stretching exercises in different positions. The rest time between each set was 1: 3 and between repetitions was 1: 1. Details of the sets and the repetitions are presented in Table 1.

### Statistical analyses

Data was visually analyzed with histograms, Q-Q plots and Kolmogorov-Smirnov tests were used to determine normality of data distribution. All data were analyzed using the Statistical Package for Social Sciences (SPSS, Version 18.0, Chicago; IL). The standard error of measurement (SEM) was used to determine the consistency of the measurements. Then, normally distributed, paired sample t tests were used to analyze the within- group differences for IR, EMG muscle activity, joint position sense, eccentric, concentric strength and ER:IR strength ratio data. Also, independent sample t tests assessed the difference in IR, EMG muscle activity, joint position sense, eccentric, concentric strength and ER:IR strength ratio between the athletes in the experimental and the control groups. A level of P < 0.05 was identified as statistically significant. The effect size (Cohen’s d) was calculated to determine the magnitude of the difference between groups and was interpreted as small if d ≤ 0.5, moderate d = 0.5–0.8 and large d>0.8 respectively [22].

## Results

Ninety males were recruited; sixty met the inclusion criteria for the study and were randomized into two groups. The experimental and control groups had a participation rate of 100% during the study (Fig. 1).

The groups were similar in age, height, body mass and exercise experience and there were no significant differences between groups at baseline, p = 0.746, p = 0.482, p = 0.759 and p = 0.638 respectively (P > 0.05) (Table 2).

**Table 2:** Demographic characteristics of participants^a^

Variables	The throwing exercise with TheraBand (n = 30)	Control (n = 30)	p
**Age (years)**	23.9 *±* 4.4	23.4 *±* 3.8	0.746
**Height (cm)**	179.8 *±* 6.5	181.3 *±* 6.9	0.482
**Body Mass (kg)**	74.2 *±* 3.6	73.2 *±* 4.7	0.759
**Exercise Experience (years)**	5.4 *±* 2.3	6.6 *±* 1.7	0.638

^a^Values are expressed as mean ± standard deviation

A significant interaction of group and time was observed following the study with selected (strength, GH joint position sense, ROM and electromyography) measures which indicated that training responses were different between groups.

Data showing the ICC, SEM and MDC for the activation (%MVC) of the intended muscles were as follows: Anterior deltoid; 0.314, 0.9, 2.5 respectively; Middle deltoid; 0.98, 1.03, 2.8 respectively; Posterior deltoid; 0.48, 0.67, 1.8 respectively, Supraspinatus; 0.40, 0.85, 2.3 respectively; Infraspinatus; 0.05, 0.68, 1.9 respectively. Also, the ICC, SEM and MDC for the onset time (ms) of the intended muscles were as follows: Anterior deltoid; 0.10, 6.94, 19.2 respectively; Middle deltoid; 0.18, 9.01, 24.9 respectively; Posterior deltoid; 0.07, 5.80, 16.09 respectively, Supraspinatus; 0.07, 5.41, 14.9 respectively; Infraspinatus; 0.01, 7.45, 20.6 respectively.

For IR ROM the ICC was 0.40, the SEM 1.41 and MDC 3.9, for eccentric strength for ER, the ICC was 0.73 and the SEM 1.57 and MDC 4.3; for concentric strength for IR, the ICC was 1.0, the SEM 1.24 and MDC: 3.4; for joint position sense, the ICC was 0.53, SEM .57 and MDC 1.5; and for functional strength ratio the ICC was 0.96 and the SEM 0.04 and MDC 1.3.

### Electromyography

**Fig. 3:**
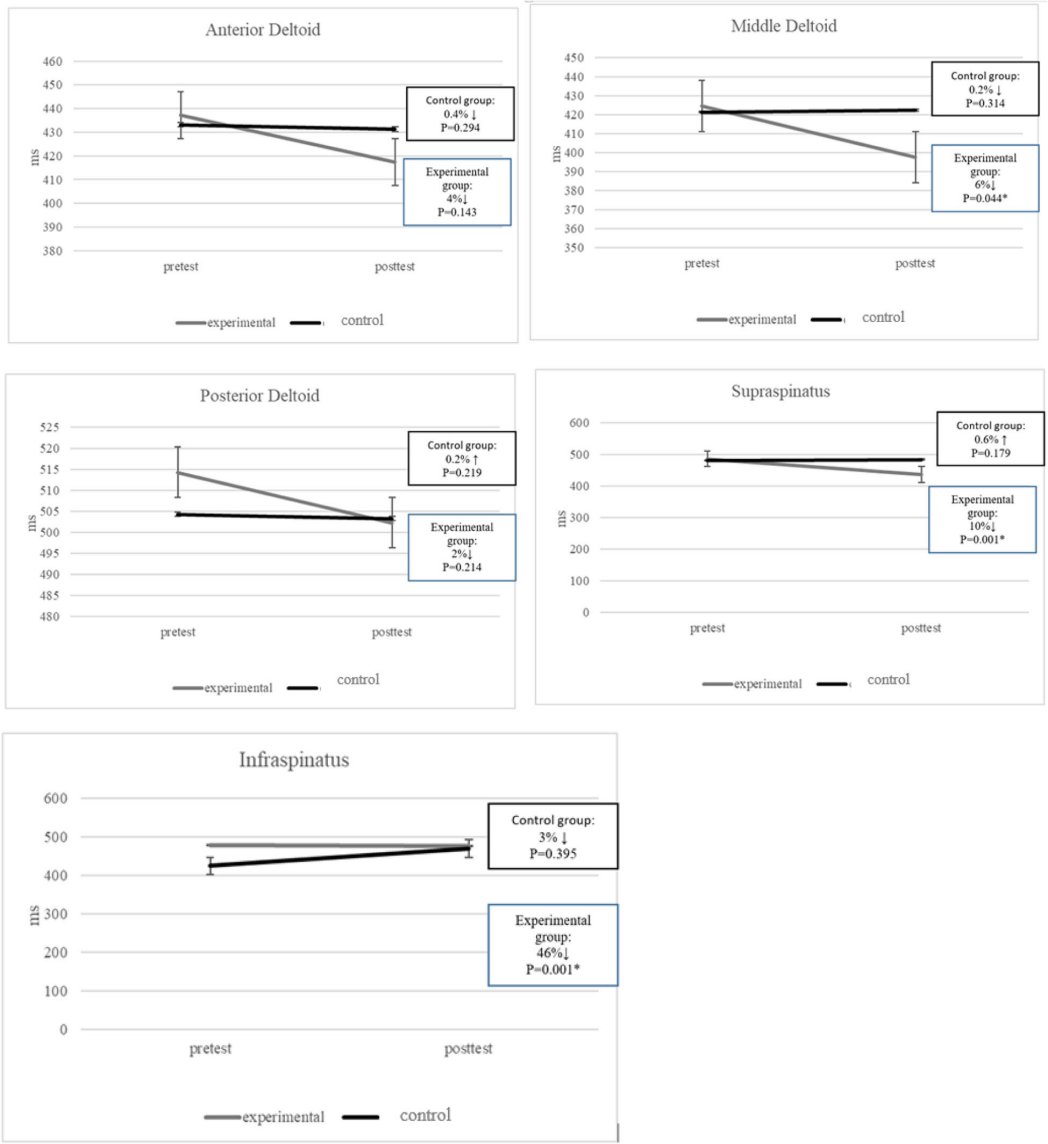
Muscle onset time (ms) alteration from pretest to post-test

A significant interaction for group and time was found following the throwing exercise with a TheraBand for the onset time of the middle deltoid (*p* = 0.044), supraspinatus (*p* = 0.000), and infraspinatus (*p* = 0.000), also in the activation of anterior deltoid (*p* = 0.008), middle deltoid (*p* = 0.008), posterior deltoid (*p* = 0.004), supraspinatus (*p* = 0.001), and infraspinatus (*p* = 0.001). Compared to the control group, the throwing exercise with a TheraBand showed a statistically significant reduction in the onset time for the middle deltoid, (95% CI, 0.11 to 1.04; *p* = 0.046; ES = 0.13), supraspinatus, (95% CI, 0.24 to 1.29; *p* = 0.001; ES = 0.44), and infraspinatus, (95% CI, 0.27 to 1.32; *p* = 0.001; 0.46), also, a statistically significant reduction in the activation of the anterior deltoid (95% CI, 0.19 to 1.24; p = 0.005; ES = 0.22), middle deltoid (95% CI, 0.17 to 1.21; *p* = 0.007; ES = 0.22), posterior deltoid (95% CI, 0.24 to 1.26; p = 0.004; ES = 0.26), supraspinatus (95% CI, 0.30 to 1.36; p = 0.001; ES = 0.32), and infraspinatus (95% CI, 0.22 to 1.38; p = 0.001; ES = 0.35) (Figs. 2 and 3).

### Shoulder IR ROM and muscle strength

**Fig. 4:**
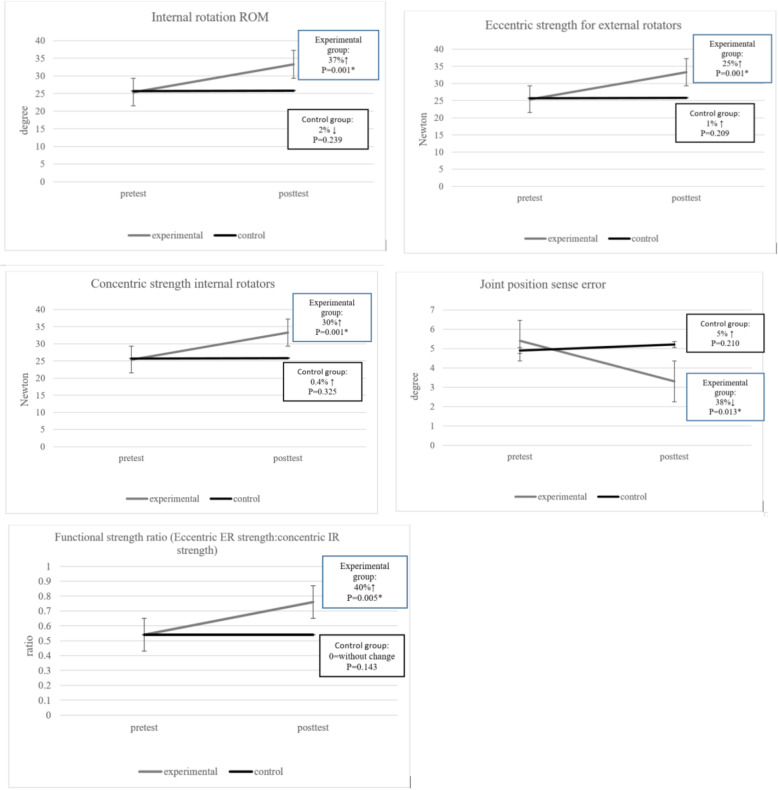
Internal rotation ROM, Eccentric strength for external rotators, Concentric strength internal rotators, Joint position sense error, and Functional strength ratio (Eccentric ER strength:concentric IR strength)

A significant interaction for group and time was found following the throwing exercise with a TheraBand program on shoulder IR ROM measure (p = 0.000), shoulder eccentric (p = 0.000) and concentric strength (p = 0.000) measures. Compared to the control group, the throwing exercise with a TheraBand showed a statistically significant improvement in the shoulder IR ROM (95% CI, 0.32 to 1.11; p = 0.001; ES = 0.55), shoulder eccentric strength (95% CI, 0.12 to 0.97; p = 0.001; ES = 0.36) and shoulder concentric strength (95% CI, 0.23 to 1.09; p = 0.001; ES = 0.43) (Fig. 4).

### ER: IR ratio and GH joint position sense

A significant interaction for group and time was also found following the throwing exercise with a TheraBand on functional strength ratio measures (p = 0.005) and GH joint position sense (p = 0.013). Compared to the control group, the throwing exercise with a TheraBand showed more statistically significant improvements in the Functional strength ratio (95% CI, 0.09 to 1.13; p = 0.021; ES = 0.75) and GH joint position sense (95% CI, 0.04 to 1.08; p = 0.033; ES = 0.65) (Fig. 4).

## Discussion

In asymptomatic male participants (volleyball players) with GIRD, the results showed statistically significant improvements in the deltoid, infraspinatus and supraspinatus muscle activations, IR ROM, ER: IR strength ratio, and GH joint position sense in the experimental group compared with the control group following the 8-week intervention.

Overhead athletes may be prone to pathologies such as shoulder internal impingement, because of the proximal force produced to resist distraction, horizontal adduction and internal rotation at the shoulder during arm deceleration [3, 9, 10, 23–27]. A previous study has suggested that stretching techniques to GH internal rotation and to the posteroinferior glenohumeral tissues help the symptoms of patients with GIRD [28]. While additional research has showed the effects of strengthening exercises on internal and external rotators on the dominant side [23] and the effects of functional exercises on eccentric rotation strength during prevention and rehabilitation programs.

### Electromyography

According to the EMG data obtained for the experimental group, this study reports statistically significant decreases in the middle deltoid, infraspinatus and supraspinatus muscle activations and timings in athletes with GIRD. While the activation significantly reduced in the anterior and posterior deltoid, the timing properties did not change. Although the activation and onset time of the mentioned muscles were statistically significant different between the two groups, most of the effect sizes were small. As a result, there were no clinically significant differences between the two groups regarding the activation and onset time of the mentioned muscles. Also, the pre to posttest differences were greater than the SEM associated with the anterior deltoid and posterior deltoid measurements (Pre-post difference ~ 18, SEM, 6.94; Pre-post difference ~ 11, SEM, 5.80 respectively), these changes could be interpreted as clinically significant. However, there were neither significant statistical nor clinical differences between the two groups in anterior (p = 0.134, ES = 0.07) and posterior (p = 0.219, ES = 0.05) onset time. Having neuromuscular dynamic control over the newly retained ROM is vital to evaluate if a treatment is successful. However, increased IR ROM through passive stretching, which is not functional, does not guarantee neuromuscular dynamic control [6].

GIRD not only effects the timing of stabilizing muscles but also the flexibility of the soft tissue surrounding the scapula and GH [1, 7, 29]. A systematic review reported that joint instability could be the consequence of altered rotator cuff muscle activity and emphasized more studies to determine the alteration of rotator cuff muscle activity in individuals with shoulder dysfunction [30]. Lin et al., 2016 reported rotator cuff and scapular strengthening exercises. However, these exercises trained the rotator cuff muscles as abductors. This method did not significantly change the activation of the deltoid, supraspinatus and infraspinatus in healthy individuals (P > 0.05). They proposed to examine the effects of this type of intervention on specific symptoms in individuals with shoulder dysfunction [15].

The present study selected strengthening exercises for retraining the rotator cuff muscles as stabilizers accompanied by stretching exercises to the posterior GH joint and soft tissues. The reduction in the timing of the shoulder muscles might primarily result from neural adaptation, especially during the earlier weeks of exercise training [31]. Improved intra- and intermuscular coordination, and increased shoulder joint mobility might be possible factors implicated in reducing the average EMG activity of these muscles in patients with GIRD [31] after the 8-week throwing exercise with the TheraBand.

### IR range of motion

Following the 8-week throwing exercise with the TheraBand, there was a statistically significant difference in shoulder IR within the experimental group (p = 0.000, SEM = 1.41, MDC = 3.9, ICC = 0.40) and between the two groups (p = 0.001, ES = 0.55), favoring the experimental group.

In a study investigating home-based treatment exercise to increase IR ROM, the proposed time for stretching was 60 min. However, other factors must be applied to prolong effects on the new IR ROM. These include low load, long duration activity during stretching, using stretching with cryotherapy and perhaps most importantly, having dynamic control over the newly gained ROM to gain neuromuscular dynamic control [6]. The lack of these mentioned procedures in the treatment of the control group could be the reason why there was no difference between the pre to posttest data after the 8-week intervention.

Bailey et al., (2017) [32], and Tucker and Slone (2016) [28] support the effect of a slipper stretch with (+ 12.1, p = < 0.001) and without (7.2, p = < 0.001) manual therapy, and proprioceptive neuromuscular facilitation for increasing IR in athletes with GIRD respectively. Previously, Maenhout et al., (2012) have reported that athletes with GIRD may have a decrease in the acromion humeral space compared to those without GIRD. They managed GIRD with stretching and reported an increase in IR and the acromion humeral space and concluded that the decrease in IR may be associated with the loss in the acromion humeral space in GIRD [33]. Moreover, Guney et al., (2015) reported that relieving the tightness of the posterior capsule by stretching, especially when accompanied by stabilizing the scapula, or GH can result in increasing IR in GIRD [13].

Physiological stretching has been recommended to restore capsular extensibility and to remodel capsular tissues [28, 32–34]. Capsular stretching, accomplished in this study also included physiological stretching, not only stretched the musculotendinous structures but indirectly stretched the various portions of the glenohumeral capsule. Also, improved strength and timing of the rotator cuff/deltoid muscle force coupling mechanism reported in the experimental group could be another factor contributing to the significant improvement in IR.

### Strength

This study showed significantly improvements in eccentric and concentric strength in ER (ES:0.36) and IR (ES:0.43) respectively, and the functional strength ratio (ES:0.75) in the experimental group. This result demonstrates the positive effects of the throwing exercise with the TheraBand on muscle strength in the experimental group. If the purpose of treatment is regaining the strength of the rotator cuff and shoulder muscles, passive and active stretching should be followed by strengthening exercises [6]. As the control group only underwent stretching exercise, increasing in muscle strengthening did not occurred in these participants.

Ozer et al. (2011) [12], and Duzgun et al. (2010) [14] reported positive effects of a rope jumping training program on the isokinetic strength of lower and upper extremity muscles, respectively. Duzgun et al., 2010 implemented the full and empty can test to evaluate the improvement of shoulder muscle strength because they believed both tests were appropriate to assess elevation in the scapular plane with the stabilizing effect on the supraspinatus muscle, which may mimic the performance of a jumping rope. They believed that this program could play a significant role in the preparation of volleyball players as special training for a kinetic-chain model.

To treat overhead athletes with GIRD, the program design should involve re-establishing the ER:IR strength ratio [10]. A study suggested to clinicians that fatigue response and the development of local muscular endurance during three sets of 15–20 repetitions in a 4-week isotonic intervention could increase 8–10% internal and external rotation strength in healthy subjects. Specifically, external rotation strengthening exercises could normalize external/internal rotation strength in overhead athletes [7]. In addition, theoretically, by stretching the muscle, overall performance may increase in activities of daily living or in sports. This may be the result of increasing the potential energy available for concentric contractions. Also, using a small towel in the axilla could prevent decreased blood flow in the supraspinatus tendon, increase the subacromial space and elevate muscular activity by 10% in the infraspinatus muscle when compared with rotational exercises performed in full adduction [7].

The throwing exercise with the TheraBand seems to affect the length of the muscles and tendons which could produce more force according to the length-tension curve theory in a muscle and improve neuromuscular coordination in agonist and antagonist muscles [10]. As a result, these changes may increase the strength of the shoulder muscles.

### Joint position sense

In this study, there was a significant change from pre to post testing for joint position sense (ICC 0.53, SEM .57 and MDC 1.5) in the experimental group. Also, a significant change was reported in the joint position sense in the participants with GIRD in the experimental group (p = 0.033, ES = 0.65) compared to the control group.

Regarding the relationship between upper and lower extremities via the kinetic chain, Ozer et al. compared the effects of a 12-week rope program with a weighted rope jumping program on joint position sense and coordination of the lower extremities in volleyball players. These authors recommend that a rope jumping training especially a weighted rope one as a basic and cost- effective activity not only modify the coordination of the lower extremities, but also balance the upper and lower extremity muscles for prevention and rehabilitation programs in volleyball players.

To be effective on a joint position sense, a treatment should retrain the neuromuscular system. To enhance joint position sense, exercise training must lead to perceptual learning and improve the capacity of sensory differentiation and signal processing in a familiar state [2, 10]. When muscles are stretched in motorcycles, the stimulation rate of the muscle spindle is more than that of the muscle in the short term, and this is closely related to the precision of the joint position sense and awareness [2]. Also, during active muscle contractions, gamma nerve activity simultaneously increases the activity of the muscle spindle and the muscles. Increasing the tensile tendency in the activated muscle spindles increases the sensitivity of the joint position sense [35].

In our study, the calculation of the effect size indicates a medium (0.50) to large (1.00) effect size for the variables, suggesting that the throwing exercise with a TheraBand in a clinical setting may selectively improve the functional strength ratio in participants with GIRD. Also, the ICC for IR ROM and joint position sense are quite low. The low ICCs suggest that the repeatability of the measurement is not as consistent as it should be especially when comparing these values to those reported in the literature.

## Limitations

Given the recommendations of the study, the authors must acknowledge some limitations. Firstly, this study investigated the effect of the intervention on asymptomatic athletes with GIRD. Future studies should investigate the effect of throwing exercise with a TheraBand on symptomatic athletes with GIRD. Secondly, we did not perform follow-up measures after the eight-week treatment to see if GIRD was progressive or regressive. Although the results obtained in the present study are promising, the extrapolation of the results to a long-term follow-up should be viewed with caution. Also, the surface EMG measurement of the supraspinatus is always challenging because it is located under the upper trapezius. In addition, the authors did not check for involvement in any sports activities of the groups during the study. Another limitation of this study is related to the technique to measure IR. In this study, the standard sleeper at 90° technique was used to measure IR ROM but recently sleeper stretch on a 45° position and a quarter turn toward the back-placing the glenohumeral joint in the scapular plane are recommended to produce the best stretch on the posterior capsule and to decrease risk of impingement [6]. Also, the technique which was used to stabilize the GH may restrict the normal arthrokinematics of the glenohumeral joint possibly resulting in the least amount of IR [6, 13]. Finally, some of the reported ICC are in the poor to fair classification range suggesting measurement consistency is questionable [36]. This is a concern and may have some influence on the results. Future studies should be sure about consistency in the quality of measurements they take.

## Conclusion

To our knowledge, we are the first to report the improvement in neuromuscular control, strength, joint position sense and functional rotator cuff ER-IR ratio in asymptomatic volleyball players with GIRD after an 8-week exercise-based intervention. We recommend the protocol be applied in clinical rehabilitation centers for volleyball players with GIRD to restore neuromuscular control.

## Data Availability

The datasets analyzed during the current study are available from the corresponding author on reasonable request.

## Abbreviations

GIRD: Glenohumeral internal-rotation deficit
IR: internal rotation
ER: external-rotator
EMG: Electromyography
ROM: range of motion
MVC: maximum voluntary contraction
